# Differing physiological performance of coexisting cool- and warmwater fish species under heatwaves in the Midwestern United States

**DOI:** 10.1371/journal.pone.0301130

**Published:** 2024-03-22

**Authors:** Qihong Dai, Cory D. Suski

**Affiliations:** 1 Program in Ecology, Evolution, and Conservation Biology, University of Illinois at Urbana-Champaign, Urbana, Illinois, United States of America; 2 Department of Natural Resources and Environmental Sciences, University of Illinois at Urbana-Champaign, Urbana, Illinois, United States of America; Benha University, EGYPT

## Abstract

Heatwaves are becoming more frequent and intensified with climate change. Freshwater ecosystems are among the most threatened, within which, differing responses between cool- and warmwater species to heatwaves can lead to fundamental changes in communities. Physiological experiments can identify potential mechanisms underlying the impacts of such heatwaves on fish communities. In the current study, we quantified the oxygen consumption rate, aerobic scope and swimming performance of cool- and warmwater fish species following the simulation of short-term heatwaves currently occurring in streams in the Midwestern United States. The coolwater predator walleye (*Sander vitreus*) showed clear thermal disadvantages relative to the warmwater predator largemouth bass (*Micropterus salmoides*), based on a high metabolic cost during the heatwave, low metabolic activity when encountering prey, and reduced swimming performance following the heatwave. Largemouth bass also showed a thermal advantage relative to the warmwater prey fathead minnow (*Pimephales promelas*) related to swimming performance and energetic costs, highlighting differing thermal responses between predators and prey. This study demonstrates the importance of considering short-term extreme thermal events in the response of aquatic communities to climate stressors.

## Introduction

Heatwaves, defined as acute, short-term increases in ambient temperature ranging from hours to days, are negatively impacting ecosystems globally, with consequences that include terrestrial forest fires and marine coral bleaching [1, 2]. Freshwater ecosystems are among the most threatened in the world, with the highest extinction rates among vertebrates [3]. In agricultural landscapes, such as the Midwestern United States, the intensity and duration of heatwaves in rivers could be further exacerbated due to reduced riparian cover, a lack of thermal refuges, and diminished groundwater recharge [4]. Such heatwaves under climate change inevitably elevate the metabolic demands of aquatic species [5]. Compared to warmwater fishes, coolwater or coldwater species could have higher metabolic costs and higher sensitivity to heatwaves (where sensitivity refers to the degree to which a species is affected by or susceptible to a climate-related change) [6], and thus be more negatively impacted by these events in terms of behavior, growth, and possibly, survival [7–9].

The differing responses of cold, cool, and warmwater fish species to climate change could fundamentally change the structure of local fish communities [10]. Under climate change, habitats may no longer provide the thermal ranges required by native species, offering opportunities for other species with a competitive advantage to become dominant [11]. For example, shifts from the dominance of the coolwater predator walleye (*Sander vitreus*) to the warmwater largemouth bass (*Micropterus salmoides*) have been projected in 91% of Wisconsin lakes by 2089 [12]. In Ontario, Canada, coolwater walleye and cisco were predicted to decline by 22 and 26%, respectively, with up to a 422% increase in warmwater smallmouth bass (*Micropterus dolomieu*) populations at the same locations [13]. Besides long-term projections, localized short-term heatwaves that are part of diel fluctuations in aquatic environments could temporarily pass the “good growth” range for coolwater species, giving warmwater species thermal advantages [14]. The short duration of such heatwaves is not expected to allow acclimation or adaptation, thus the performance of fishes experiencing heatwaves heavily relies on transitory physiological and behavioral responses [15]. For fish communities where cool- and warmwater species coexist now, the influence of climate change, especially short-duration heatwaves associated with diel temperature fluctuations, could be the deterministic mechanism that ultimately reshapes the composition of local fish communities [16].

Predator-prey relationships in local fish communities can also be altered under climate change by limitations to the thermal performance of fishes under altered thermal regimes. Indeed, the temperature dependence of predation success could be a key factor determining the relative performance of predators and prey. Higher temperatures could impact the attack speed of coolwater predators, but not the escape speed of warmwater prey [12], causing certain thermal thresholds to become a tipping point for predator-prey interactions that shift community composition. Besides behavior, temperature also affects energy gain (ingestion) and loss (metabolism). The temperature-dependent performance of ingestion and metabolism may differ across trophic levels, further perturbing the stability of local communities [17].

Large-scale ecological modeling can identify potential trajectories for fish community changes under different climate scenarios [12], but smaller-scale physiological experiments help verify or reject such ecological projections by identifying putative mechanisms by which fishes respond to climate change, especially heatwaves. The sensitivity of an individual to thermal stressors can be partially explained by the oxygen- and capacity-limited thermal tolerance (OCLTT) hypothesis [18], despite its limitations [19, 20]. The OCLTT postulates that, during periods of increased temperature, the demand for oxygen in tissues increases until it exceeds cardiorespiratory capacity, causing a loss of performance [21, 22]. Compared to warmwater species, coolwater fishes may experience a decline or plateau in their maximum metabolic rate at a lower temperature than warmwater fish, thus having impairment or loss of performance earlier during warming [21, 23, 24]. At the individual level, behavioral changes, particularly swimming performance, can be a key factor in determining prey capture, predator avoidance, and migration of different species [25]. During warming, coolwater species may exceed their thermal optimum for swimming while warmwater species may be less impacted. Differences in swimming performance can alter the balance in local fish communities by influencing survival and fitness [26]. With an improved understanding of physiological mechanisms, we can better predict and explain the trajectories of potential changes to aquatic communities.

Under climate change with heatwaves, differing responses between cool- and warmwater species could drive changes in fish communities. To define potential mechanisms for change in Midwestern fish communities in the future, we focused on three species that can be found in lentic environments, are both ecologically and recreationally important, and have long-term sampling records in the Midwestern United States: 1) largemouth bass, one of the most abundant warmwater predator species; 2) walleye, a popular coolwater predator species that is becoming less abundant, coexisting with largemouth bass; and 3) fathead minnow (*Pimephales promelas*), a widespread and ecologically important prey species that co-occurs with both predators [27, 28]. We quantified differences in physiological performance of 1) walleye vs. largemouth bass as predators and 2) fathead minnow vs. both predators at summer temperature with short-duration heatwaves in the laboratory. The physiological performance measures included whole-organism maximum and standard oxygen consumption rates ($\dot{M}O_{2}$), aerobic scope, $\dot{M}O_{2}$ during heatwaves and predator-prey interactions, and critical swimming speed (U_crit_) for each species. The duration and magnitude of heatwaves was based on *in situ* monitoring of forested streams in the Kaskaskia River Watershed, Illinois, USA, where daily maximum temperatures of streams can reach 30° C during the day, and then cool down again after the sun sets (see Fig 1 from [4]). Compared to warmwater largemouth bass and fathead minnow, we predicted that coolwater walleye would show reduced performance following expossure to summer conditions, even in the absence of a heatwave, in terms of the narrowest aerobic scope, a limited metabolic response during predator-prey interactions, and impaired U_crit_. Quantifying and comparing the physiological responses of cool vs warmwater species under climate change with heatwaves could offer mechanistic evidence of how and why fish communities could change in the future, guiding management practices for freshwater ecosystems under climate change.

**Fig 1:**
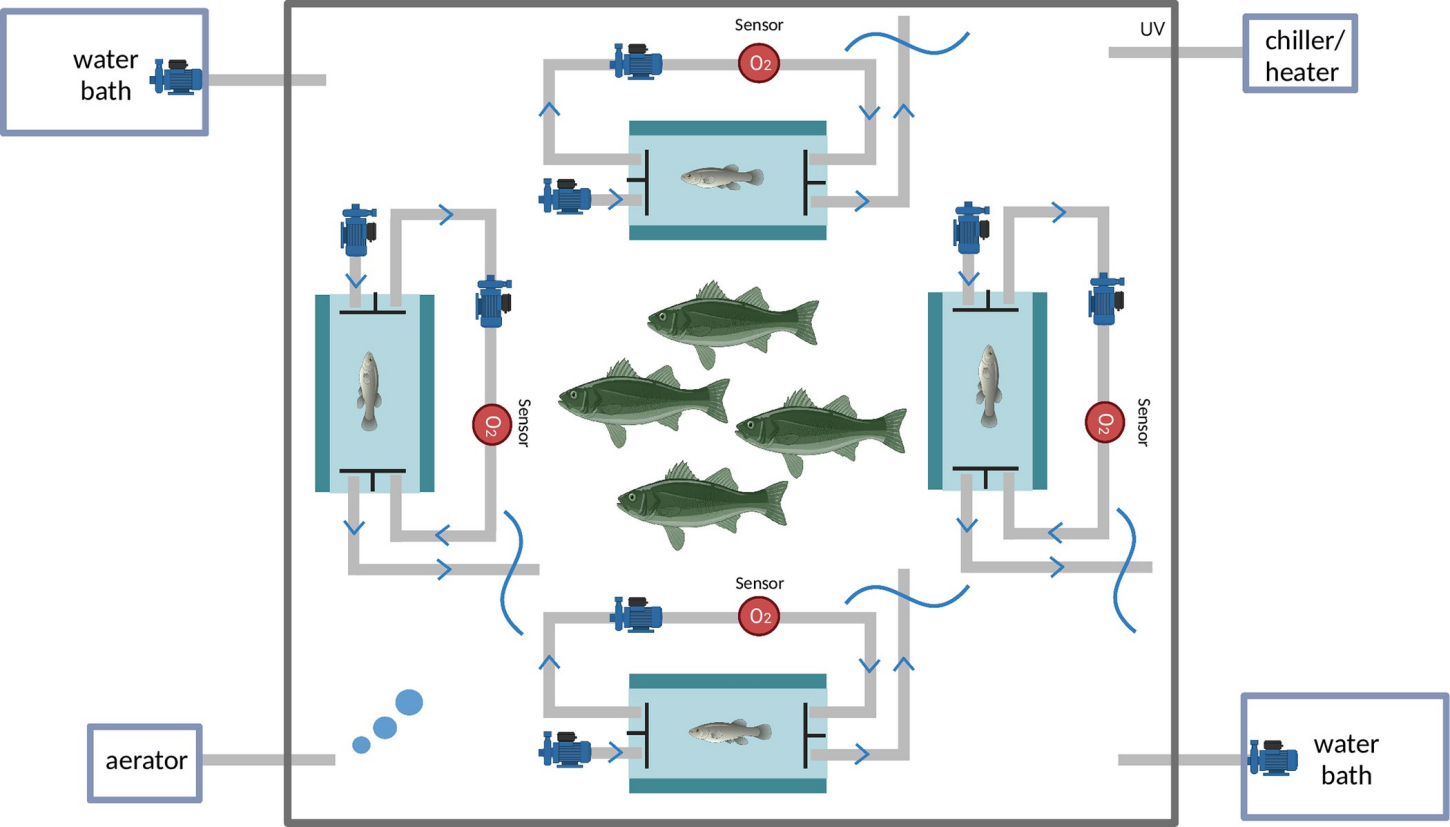
Diagram of intermittent respirometry settings. While testing prey in respirometry chambers, four predator individuals (largemouth bass (*Micropterus salmoides*) or walleye (*Sander vitreus*)) were gently introduced to swim freely around respirometry chambers in the tank to ensure both visual and olfactory cues. While testing predators in chambers, 20 fathead minnow (*Pimephales promelas*) were gently introduced into four mesh cages (five in each cage) right above the four respirometry chambers, plus another 20 free swimming fathead minnow in the tank.

## Materials and methods

### Fish collection and husbandry

Laboratory experiments were conducted from May 2021 to December 2021, using fathead minnow acquired from Anderson Minnows Farm (Lonoke, AR, USA), largemouth bass acquired from Jake Wolf Memorial Fish Hatchery (Topeka, IL, USA), and walleye acquired from Keystone Hatcheries (Richmond, IL, USA). Fish were housed in static tanks at the Aquatic Research Facility at the University of Illinois Urbana-Champaign. Before fish arrival, all holding tanks were cultured with nitrifying bacteria using Fritz Turbo Start 700 (Fritz Aquatics, Mesquite, TX, USA), coupled with ammonium chloride, for up to three weeks. Dissolved oxygen (DO) in all tanks was maintained at > 90% saturation and a ultraviolet (UV) filter was used to minimize the growth of bacteria and algae.

The holding of fish in the laboratory was similar across the different species. More specifically, upon arrival at the research facility, fathead minnow (total length 59 ± 5 (standard deviation) mm and total weight 1.8 ± 0.5 g for individuals used in the experiments described below) were distributed randomly into five 110 L tanks, with a maximum density of four fish 10 L^-1^. Fathead minnow used as food for predator species (i.e., largemouth bass and walleye) were distributed randomly into another five 110 L tanks, with a maximum density of six fish 10 L^-1^. Juvenile largemouth bass (n = 50, total length 236 ± 11 mm and total weight 210 ± 34 g for individuals used in experiments) were evenly distributed in four 567 L tanks. Juvenile walleye (n = 50, total length 174 ± 20 mm and total weight 49 ± 20 g for individuals used in experiments) were evenly distributed in three 567 L tanks. Fathead minnow were fed to satiation daily with fish flakes and brine shrimp (*Artemia artemiidae*). To ensure predator species became familiar with fathead minnow as prey, both largemouth bass and walleye were fed with at least three fathead minnow per individual every two days. Before any experiments described below, fathead minnow were fasted for at least 24 h, while largemouth bass and walleye were fasted at least for 48 h. Water temperatures in holding tanks were initially set to match shipping temperatures, and then were increased 1°C per day until reaching 25°C (TK-500; TECO, Ravenna, Italy) to mimic average summer water temperatures in the Kaskaskia River Watershed based on temperature records in 2020 [4] and Illinois stream gauge monitoring stations [https://dashboard.waterdata.usgs.gov/app/nwd/en/?aoi=default, accessed June 13, 2023]. The acclimation period at 25°C lasted at least two weeks prior to the start of any experiment [29–31]. Throughout the entirety of the study, fish were only used once, and no individual was used in more than one aspect of experiment.

#### Maximum and standard oxygen consumption rates (MMR and SMR)

To quantify maximum and standard whole-organism oxygen consumption rate ($\dot{M}O_{2}$, mg O_2_ h^-1^) of both prey (i.e., fathead minnow) and predators (i.e., largemouth bass and walleye) at summer temperature (25°C), intermittent-flow respirometry was used (Loligo Systems, Viborg, Denmark) [19]. In the system, small four glass cylindrical chambers (inner diameter: 45 mm; length: 145 mm; volume including tubing: 345 cm^-3^) were used when testing prey species, and four large glass cylindrical chambers (inner diameter: 140 mm; length: 500 mm; volume including tubing: 7697 cm^-3^) were used for large predator species. Chambers were submerged in a 440 L aerated holding tank (length: 1100 mm; width: 1000 mm; height: 400 mm) in a quiet room, with aerated water temperature controlled at 25 ± 0.2°C. A UV sterilizer (9 Watt, Pond Boss; West Palm Beach, FL, USA) minimized bacterial respiration during trials [32].

Following the completion of pilot trials, the measurement cycle for all three species was the same: a 5 min flush, 1 min wait, and 14 min measure period, resulting in 20 min cycle to ensure the concentration of dissolved oxygen remained above 80% during the measurement period while obtaining high R^2^ readings for data. A mixing pump (Universal 300; Eheim, Deizisau, Germany) connected by vinyl tubing ran to move water through the chamber and around an external circuit of gas-tight tubing to avoid any oxygen gradients. A flush pump connected by vinyl tubing connected to each chamber (Universal 300; Eheim, Deizisau, Germany) and controlled by AutoResp 2.2.2 (Loligo Systems, Viborg, Denmark) allowed for the exchange of fresh aerated water from the tank during each 5 min flush period. During the 14 min measurement period, DO in the chambers was monitored every sec using a 4-channel Minisensor Oxygen Meter with associated sensors (PreSens; Regensburg, Germany) (Fig 1). All chambers were scrubbed and rinsed before every trial, and a UV sterilizer was used constantly to minimize background respiration. The final $\dot{M}O_{2}$ accounted for background bacterial respiration by measuring respiration for a 20 min cycle before and after trials and assuming a linear increase in background respiration over time. We used the same sized chambers for both largemouth bass and walleye despite the total weight of walleye being < 50% that of largemouth bass to ensure consistency in experimental conditions for both predators and to minimize behavioral disturbances for walleye; this difference in fish size did not impact data quality.

To quantify the maximum oxygen consumption rate (MMR) for fathead minnow, largemouth bass, and walleye, we first gently introduced individuals into a 88 L circular arena (radius: 280 mm; height: 360 mm) at 2 pm. Fish were then manually chased by a dip net for 3 min or until exhaustion, quantified as being unable to show burst swimming over 30 s after preliminary trials [33]. After this chasing period, we immediately measured fish for total mass and total length, then introduced them into the respirometry chamber (during the flush cycle) without intentional air exposure treatment [33]. Once the chamber was sealed, we manually switched from the flush cycle to the 1 min wait cycle to start the measurement period. The maximum $\dot{M}O_{2}$ generated throughout the initial measurement cycle was considered to be MMR. Fish were then left undisturbed overnight until 12 pm the next day, generating a 22 hour measurement period. We used the q0.1 method to calculate standard oxygen consumption rate (SMR) [34]. Differences between MMR and SMR for each species were calculated as both absolute aerobic scope (AS) (i.e., MMR-SMR) and factorial aerobic scope (FAS) (i.e., MMR/SMR), respectively, as these two metrics can often provide different results, such that including both can increase confidence in findings [18].

### Heatwave exposure

The magnitude and duration of heatwaves used in this study were defined following the integration of a number of different considerations. For example, temperature loggers placed in the Kaskaskia River Watershed [4], Illinois, USA, showed that the highest daily temperature in July 2020 was 29.7°C for streams in forested watersheds. Walleye are considered a coolwater species (relative to warmwater species such as largemouth bass and fathead minnow) [35], and the chronic and acute thermal tolerances of walleye in the summer have previously been reported as 25 and 30°C, respectively [36]. Thus, the heatwave intensity used was set at 30°C to avoid immediate mortality of walleye, but to mimic maximum summer temperatures observed in forested streams in the Kaskaskia River Watershed. To accurately quantify the effects of short-term heatwaves on fish during laboratory simulations, the duration of the 30°C heatwave was maintained for 1 h, coupled with a 1 h ramping period from 25°C and a 1 h cooling period back to 25°C. This temperature change was achieved by pumping water from the circular arena and heating it externally using stainless steel coils placed in a hot water bath, coupled with heaters/chillers (TK-500; TECO, Ravenna, Italy) for finer adjustments. The warmed water was returned to the holding tank, and this continued until the target temperature of 30°C was achieved. While this heating was occurring, the 20-minute respirometry cycles continued to run (5 min flush, 1 min wait, and 14 min measure period) and warmed water was drawn from the circular arena into the respirometry chambers during the flush period resulting in a heat exposure for fish. This protocol for generating a heatwave mimics the natural periodicity in temperatures seen in aquatic environments in the Midwestern United States whereby a peak temperature is reached, typically in the afternoon, and the water then begins to cool as the sun sets [4]. This rate of temperature change is faster than present-day field data (i.e., 1 h vs 2–4 h) as we sought to mimic more intense heatwaves predicted in the future [4]. We also pumped water from the circular arena through stainless steel coils while the external water baths were turned off to serve as a control during the 25°C treatment and quantify any changes in metabolic rate due to laboratory holding or the presence of experimental personnel.

#### Oxygen consumption rate ($\dot{M}O_{2}$) with the heatwave and predator-prey interactions

The methods used to quantify $\dot{M}O_{2}$ during predator-prey interactions following heatwaves was similar to the MMR/SMR procedure described above. Specifically, fathead minnow, largemouth bass, or walleye were first collected from holding tanks, measured for total mass and total length, gently introduced into respirometry chambers at around 2 pm, and then left undisturbed until around 9 am the next morning. Resting $\dot{M}O_{2}$ (RMR) was calculated as the lowest 10% of measurements taken throughout this overnight period [32]. Then, for fish experiencing the heatwave treatment, water temperature in the respirometry tank was increased rapidly from 25 to 30°C within 1 h, maintained for 1 h, then cooled back to 25°C within 1 h at around 12 pm as described above. For the control group, the temperature in the circular arena was held at 25°C, and these control fish received all the same procedures.

At around 1 pm, we introduced either prey or predators into the circular arena that contained respirometry chambers to quantify the metabolic response of fish to either predators or prey during a 2 hour exposure period (Fig 1). When fathead minnow were held in respirometry chambers, this means we either introduced either four largemouth bass or four walleye into the circular arena and let them swim freely around the four respirometry chambers (Fig 1). This ensured fathead minnow, as prey, could receive both visual and olfactory cues from predators during the interaction period as water was drawn from the circular arena into the respirometry chambers during each flush period. When walleye or largemouth bass were held in the respirometry chambers, we introduced 20 fathead minnow into four mesh cages (five in each cage) into the circular arena right above the four respirometry chambers, plus another 20 free swimming fathead minnow in the circular arena holding respirometry chambers. This ensured walleye or largemouth, as predators, could receive both visual and olfactory cues from prey during the interaction period. Eight fish were measured for each treatment. All chambers were deeply cleaned with bleach before every trial, and a UV sterilizer was used constantly to minimize background respiration. Final $\dot{M}O_{2}$ accounted for background bacterial respiration by measuring respiration for 20 min cycle before and after trials and assuming a linear increase in background respiration over time.

#### Swimming performance (U_crit_) with heatwaves

Tests of critical swimming speed (U_crit_) were performed in a 5 L (30 × 7.5 × 7.5 cm test section) flow-controlled swim tunnel for fathead minnow, and a 30 L (46 × 15 × 15 cm test section) flow-controlled swim tunnel for largemouth bass and walleye (Loligo; www.loligosystems.com). Swim tunnels were calibrated using a flow meter (HFA; Höntzsch) to convert motor speed to water velocity (cm s^-1^). For these tests, individual fish were gently introduced into the swim tunnel at 25 ± 0.2°C, with water velocity at either 1 body length (BL) s^-1^ for fathead minnow, or 0.5 BL s^-1^ for largemouth bass or walleye. Right after sealing the swim tunnel, the short-duration heatwave simulation commenced using procedures identical to those described above (i.e., 1 h ramping, 1 h at 30°C, and 1 h cooling), doubling as an acclimation period to swim tunnel. Water temperature in the control group was maintained at 25°C for this 3 h acclimation period. This 3 h acclimation duration was determined following preliminary studies that showed that, after 3 h of being in respirometry chambers, $\dot{M}O_{2}$ declined to be close to SMR. Following the heatwave, water velocity was increased by 0.5 BL s^-1^ every 5 min [37] for fathead minnow, or every 20 min for largemouth bass and walleye [38], until fish became exhausted, determined when the fish failed to move off the rear screen of the chamber for > 5 s. Once exhausted, the fish was gently removed from the swim tunnel and measured for total mass and total length. Eight fish were measured for each treatment.

Critical swimming speed (U_crit_) was calculated as:

$$U_{crit}=U+\left(\frac{t}{T}\right)\times \Delta U$$

where *U* (cm s^-1^ or BL s^-1^) is the highest water velocity at which fish continued swimming for the full 5 min for fathead minnow or 20 min for largemouth bass or walleye, *ΔU* is the velocity increment (i.e. 0.5 BL s^-1^), *t* (min) is the time fish swam during the final increment, and *T* is the time increment (i.e. 5 or 20 min) [39]. Solid blocking correction was performed for largemouth bass, but not for fathead minnow or walleye, because the area of fathead minnow and walleye relative to the cross-sectional area of swim tunnels was < 5%, thereby minimizing the impact of the fish on water velocity in the tunnel [40]. This study was carried out in strict accordance with the recommendations in the Guide for the Care and Use of Laboratory Animals of the National Institutes of Health. The protocol was approved by the Institutional Animal Care and Use Committee of the University of Illinois (Protocol Number: 21063).

### Data analysis

All data were analyzed using R 4.0.2 [41] with α = 0.05. Oxygen consumption rates ($\dot{M}O_{2}$) for each individual was calculated as the slope of each 14 min measurement period obtained from the linear regression between the changes in oxygen concentration over time (mg O_2_ h^-1^), then corrected for the volume of the respirometry chamber and total mass of the fish. $\dot{M}O_{2}$ with r^2^ < 0.9 were filtered out for quality control [42].

To compare MMR and SMR of each species, linear mixed models were run using the lme4 [43] and lmerTest [44] packages, with log($\dot{M}O_{2}$) as the dependent variable, and the type of $\dot{M}O_{2}$ (MMR or SMR) and log(total weight) as the fixed factors. Fish ID was included as a random factor because multiple $\dot{M}O_{2}$ were collected from each fish. Other random factors, including test date and total length, were initially added to models, but were later removed based on higher AIC values (AIC of models with and without test date and total length, respectively: Fathead minnow: 19.95 vs.16.32; Largemouth bass: -5.27 vs. -8.49; Walleye: 6.81 vs. 2.81.). Model assumptions of linearity, normality, and homogeneity of residuals were confirmed by inspecting plots of model residuals versus fitted values and quantile-quantile plots. Marginal *r*^*2*^ values that quantify model variance with fixed factors only, and conditional *r*^*2*^ values that quantify variance for the entire model, were calculated using the MuMIn package [45].

To quantify the effects of short-duration heatwaves and predator-prey interactions on $\dot{M}O_{2}$ for each species, linear mixed models were again run, with log($\dot{M}O_{2}$) as the dependent variable, treatment (heatwave or no heatwave), time (RMR, 1 h heatwave, 1 h after heatwave, and first hour during predator-prey interaction), and log(total weight) as fixed factors, The interactions between treatment and time were also included, with fish ID as a random factor to account for potential non-independence of data (i.e. different time periods of $\dot{M}O_{2}$ for each fish). Other random factors were initially included in full models including chamber number (nested within treatment) and test date (nested within treatment) but these factors were later removed based on higher AIC values. (AIC of models with and without other random factors, were respectively: Fathead minnow with predator largemouth bass: 56.97 vs. 54.65; Fathead minnow with predator walleye: 70.74 vs. 68.71; Largemouth bass with prey fathead minnow: -13.60 vs. -17.60; Walleye with prey fathead minnow: 13.55 vs. 9.72.) Assumptions of linearity, normality, and homogeneity of residuals were again confirmed as described above. Tukey’s HSD post hoc analyses were performed to separate means using the emmeans package [46]. Marginal *r*^2^ and conditional *r*^2^ were also calculated.

To quantify the effects of short-duration heatwaves on swimming performance for each species, linear mixed models were again used, with U_crit_ (cm s^-1^ or BL s^-1^) as the dependent variable and treatment (heatwave or no heatwave) as the fixed factor. Total length (nested within treatment) was included as a random factor. Other random factors were initially included in full models but later removed based on higher AIC values, including test date nested within treatment and total mass nested within treatment (AIC of models with and without other random factors, respectively: Fathead minnow: 106.48 vs. 104.48 (cm s^-1^) and 56.54 vs. 54.54 (BL s^-1^); Largemouth bass: 111.91 vs. 110.06 (cm s^-1^) and 24.04 vs. 22.20 (BL s^-1^); Walleye: 105.62 vs. 102.44 (cm s^-1^) and 23.25 vs. 20.84 (BL s^-1^)). Assumptions of linearity, normality, and homogeneity of residuals were again confirmed as described above.

## Results

### MMR and SMR

**Fig 2:**
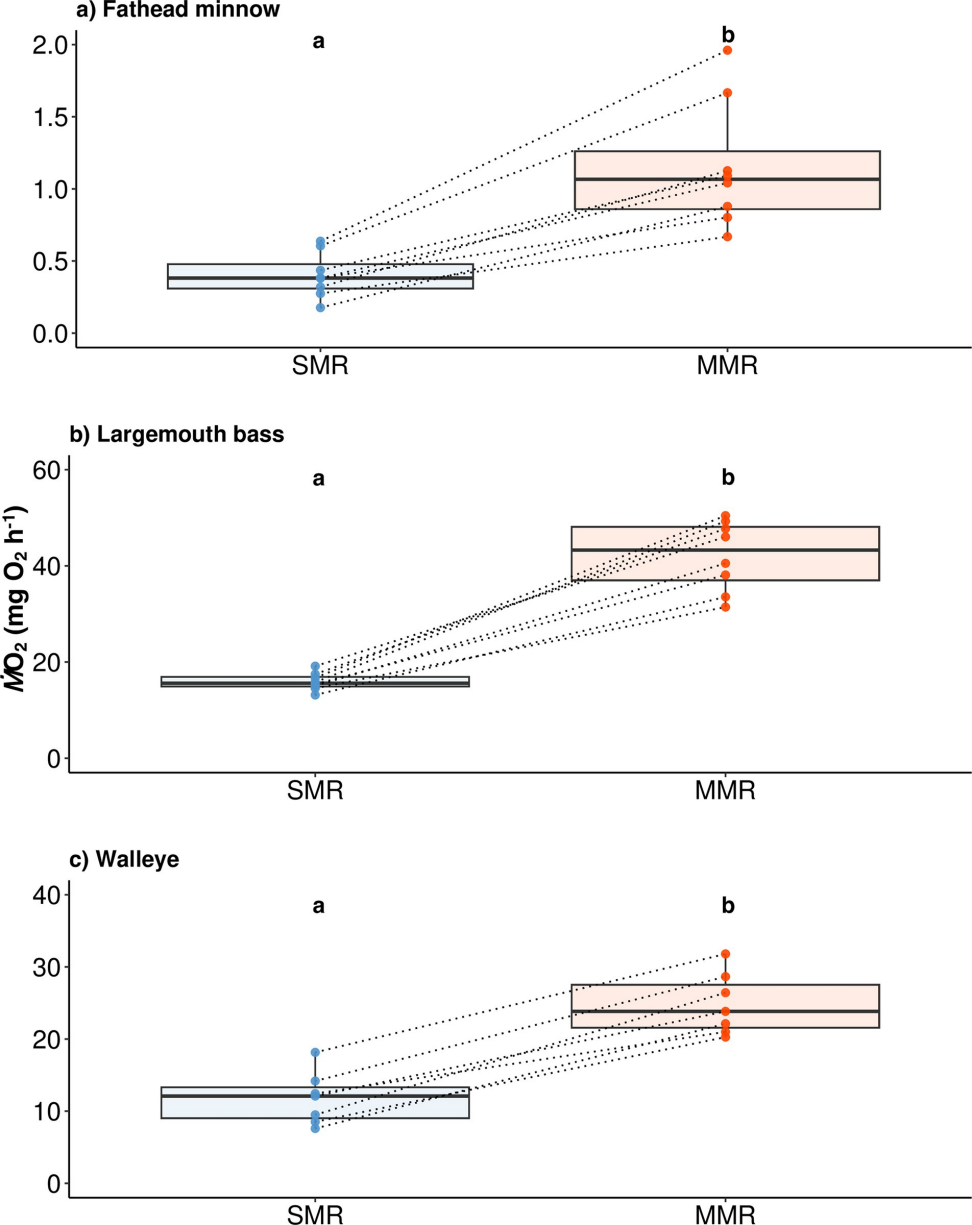
Standard and maximum metabolic rate (SMR and MMR, ± se) of fathead minnow (*Pimephales promelas*), largemouth bass (*Micropterus salmoides*), and walleye (*Sander vitreus*). Fish were held at 25°C. Groups (n = 8 per group) with different letters are significantly different (*p* < 0.05) from SMR and MMR. Dotted lines joining SMR and MMR connect data points for individual fish.

**Fig 3:**
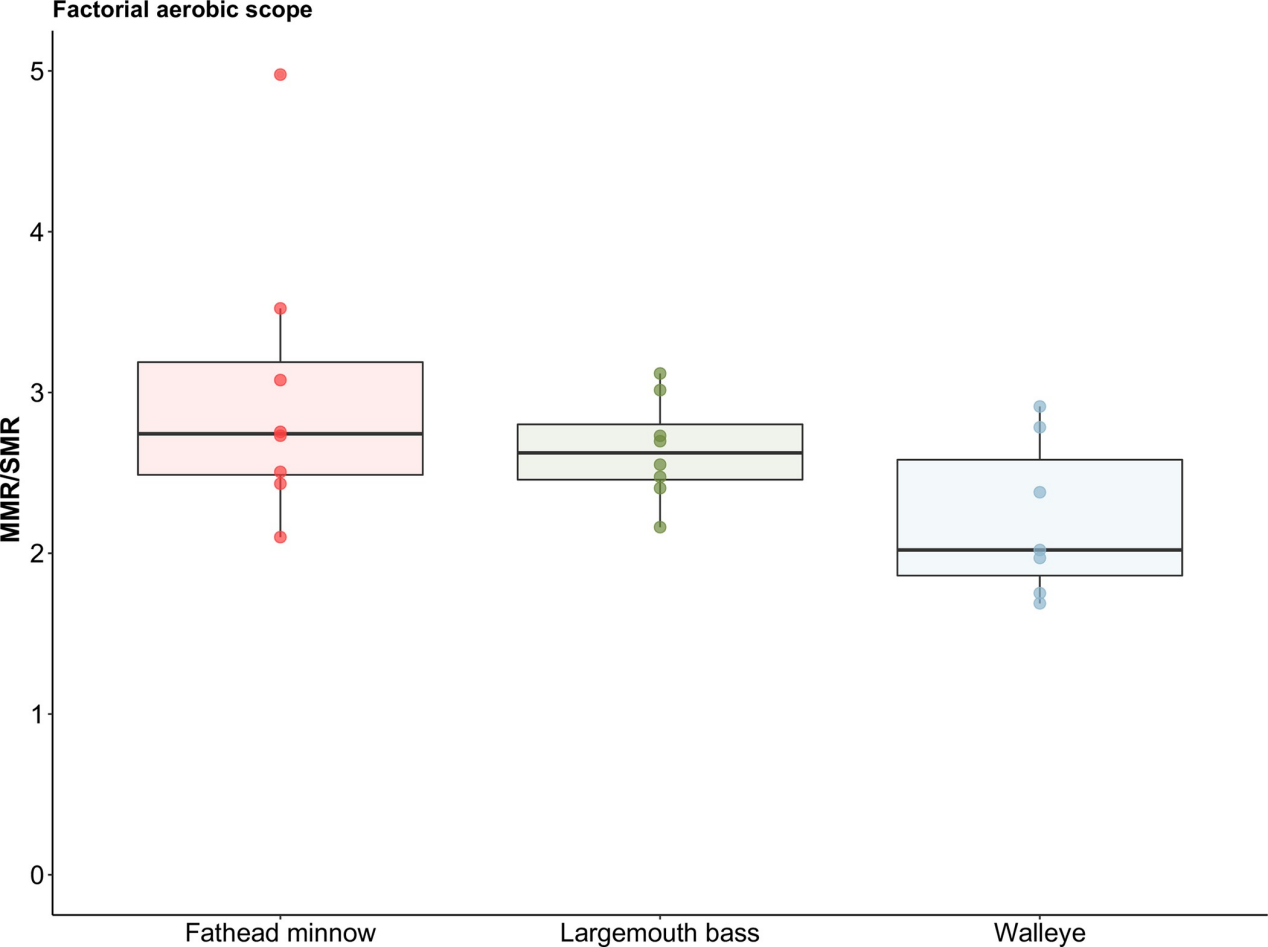
Factorial aerobic scope (maximum metabolic rate / standard metabolic rate) of fathead minnow (*Pimephales promelas*), largemouth bass (*Micropterus salmoides*), and walleye (*Sander vitreus*). Fish were held at 25°C (n = 8 per group).

After ≥ two weeks acclimation at 25°C, the different fish species showed pronounced differences in MMR. More specifically, whole-animal MMR for fathead minnow was 188% higher than SMR after two weeks acclimation at 25°C, (F_1, 7_ = 129.29 *p* < 0.01, R^2^_m_ = 0.77, R^2^_c_ = 0.91), leaving AS as 0.75 ± 0.31 mg O_2_ h^-1^ (mean ± sd) and FAS as 2.9 (Table 1 and Figs 2A and 3). For largemouth bass, whole-animal MMR was 165% higher than SMR (F_1, 7_ = 519.30, *p* < 0.01, R^2^_m_ = 0.95, R^2^_c_ = 0.97), leaving AS as 26.23 ± 6.09 mg O_2_ h^-1^ and FAS as 2.6 (Table 1 and Figs 2B and 3). For walleye, whole-animal MMR was only 110% higher than SMR (F_1, 11_ = 99.31, *p* < 0.01, R^2^_m_ = 0.89, R^2^_c_ = 0.89), leaving AS as 13.09 ± 2.68 mg O_2_ h^-1^ and FAS as 2.1 (Table 1 and Figs 2C and 3). Trends for mass-specific metabolic rates were similar to whole-animal metabolic rates (Table 1).

**Table 1:** Mean (± sd) whole-animal and mass-specific metabolic rates ($\dot{M}O_{2}$) and body size during respirometry for fathead minnow (*Pimephales promelas*), largemouth bass (*Micropterus salmoides*), and walleye (*Sander vitreus*). SMR refers to standard metabolic rate, MMR refers to maximum metabolic rate, and AS refers to aerobic scope.

					Whole animal (mg O_2_ h^-1^)			Mass specific (mg O_2_ kg^-1^ h^-1^)		
Species	Temperature (°C)	n	Total length (mm)	Total mass (g)	SMR	MMR	AS	SMR	MMR	AS
Fathead minnow	25	8	59 ± 6	1.7 ± 0.5	0.40 ± 0.16	1.15 ± 0.44	0.75 ± 0.31	231 ± 76	673 ± 208	442 ± 160
Largemouth bass	25	8	225 ± 11	188.3 ± 28.8	15.91 ± 1.86	42.14 ± 7.31	26.23 ± 6.09	85 ± 10	225 ± 29	139 ± 25
Walleye	25	7	171 ± 19	49.1 ± 17.2	11.78 ± 3.66	24.87 ± 4.27	13.09 ± 2.68	244 ± 47	526 ± 72	283 ± 77

#### $\dot{M}O_{2}$ with the heatwave and predator-prey interactions

**Fig 4:**
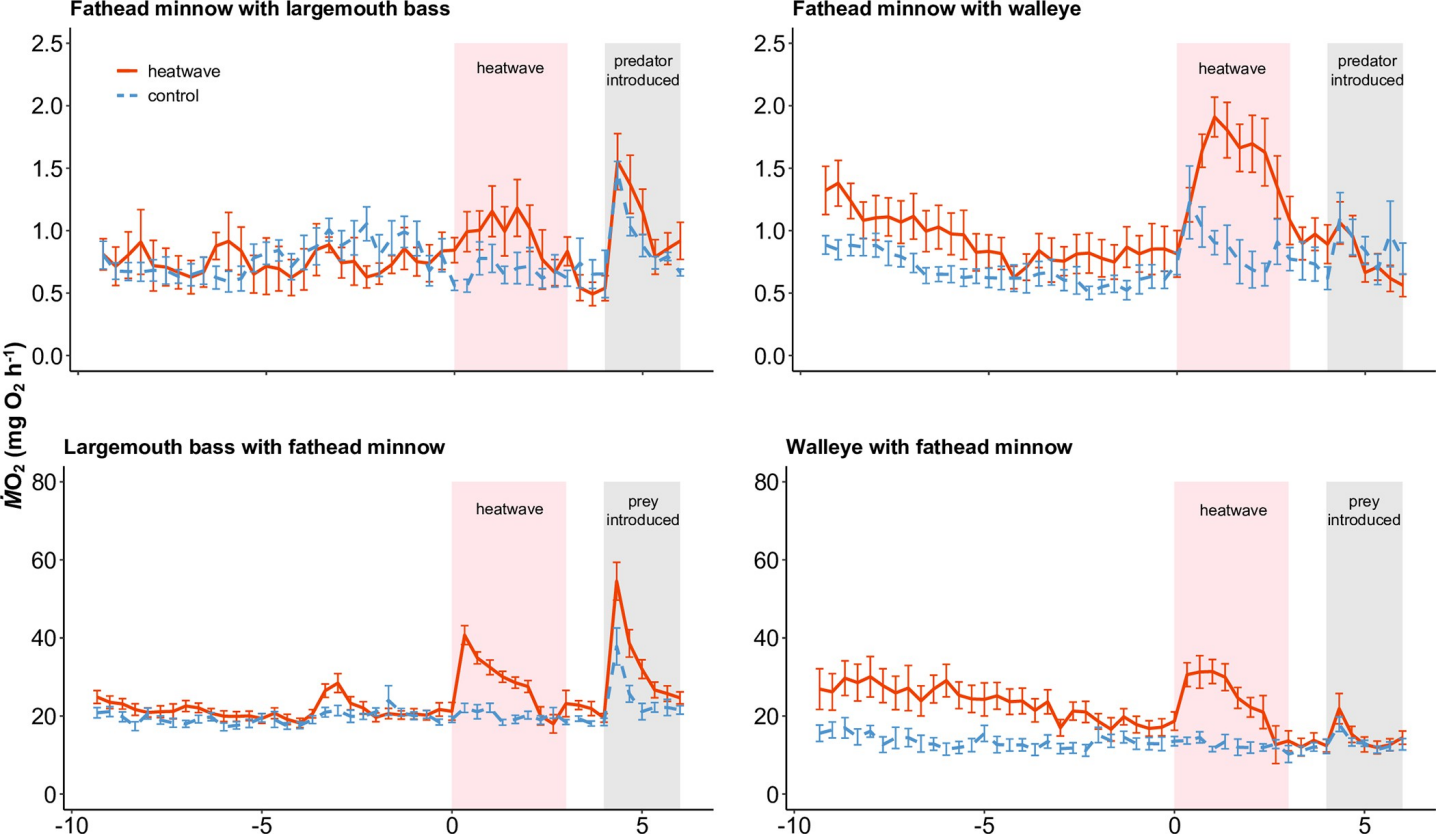
Oxygen consumption rates ($\dot{M}O_{2}$, ± se) of fathead minnow (*Pimephales promelas*), largemouth bass (*Micropterus salmoides*), and walleye (*Sander vitreus*). Measurements occurred during the heatwave (pink background) and predator-prey interaction (grey background) (n = 8 fish per group). Time at 0 h represents the start of temperature changes. The heatwave consisted of 1 h heating, 1 h of elevated temperature, and 1 h cooling.

**Fig 5:**
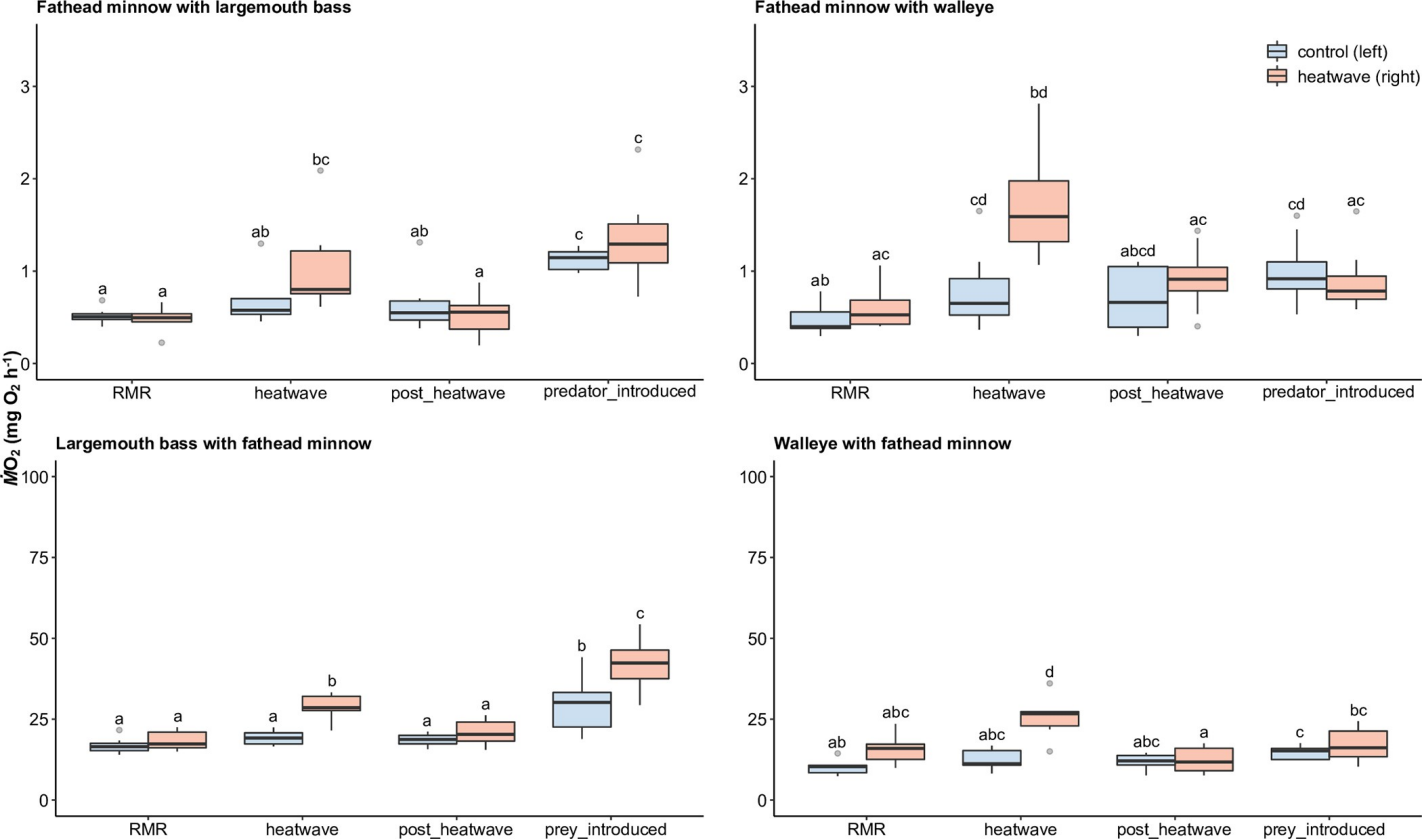
Oxygen consumption rates ($\dot{M}O_{2}$, ± se) of fathead minnow (*Pimephales promelas*), largemouth bass (*Micropterus salmoides*), and walleye (*Sander vitreus*) (n = 8 in per group) during the heatwave and predator-prey interaction. Horizontal line = median; box = first quartile to third quartile; vertical line = 1.5 interquartile range. Different letters represent statistical differences (*p* < 0.05).

The warmwater prey species, fathead minnow, rapidly increased $\dot{M}O_{2}$ during the 1 h heatwave, and showed elevated $\dot{M}O_{2}$ when encountering largemouth bass, but not walleye (Table 2 and Figs 4 and 5). During the heatwave, fathead minnow increased $\dot{M}O_{2}$ by 70% compared to 25°C. This $\dot{M}O_{2}$ during the heatwave was more than double the overnight RMR. The $\dot{M}O_{2}$ (1.07 ± 0.51 mg O_2_ h^-1^) during the heatwave was 93% of MMR (1.15 ± 0.44 mg O_2_ h^-1^). After the heatwave, the $\dot{M}O_{2}$ of fathead minnow returned to RMR. However, once largemouth bass were introduced into the tank, fathead minnow showed a significant $\dot{M}O_{2}$ increase, with a 90% increase in $\dot{M}O_{2}$ compared to the “post-heatwave” period for fish not experiencing heatwave before or more than double the increase of fish experiencing heatwave before. Such increase in $\dot{M}O_{2}$ (0.86 ± 0.53 mg O_2_ h^-1^) almost took up the entire AS (0.75 ± 0.31 mg O_2_ h^-1^). In contrast, the introduction of another predator species, walleye, resulted in no significant $\dot{M}O_{2}$ response for fathead minnow relative to the post-heatwave period.

**Table 2:** Results of linear mixed effect models examining factors affecting whole-animal metabolic rate (log($\dot{M}O_{2}$)) under heatwave and predator-prey interactions for fathead minnow (*Pimephales promelas*), largemouth bass (*Micropterus salmoides*), and walleye (*Sander vitreus*). Related to Fig 3.3-3.4, time represents different measurement periods, including resting metabolic rate (RMR), 1 h heatwave at 30°C (heatwave), 1 h post heatwave (post-heatwave), and first 1h of predator-prey interaction (interaction). Models include fish ID as a random factor. Marginal r^2^ = 0.60, 0.54, 0.82, and 0.82, while conditional r^2^ = 0.79, 0.77, 0.84, and 0.82 for fathead minnow with largemouth bass predator, fathead minnow with walleye predator, largemouth bass with fathead minnow prey, and walleye with fathead minnow prey, respectively. Significant factors are shown in bold.

Species		Sum Sq	Mean Sq	NumDF	DenDF	F value	*P*
Fathead minnow with largemouth bass predator	Heatwave or not	0.02	0.02	1	12.18	0.42	0.53
	**Time**	**7.49**	**2.50**	**3**	**36.57**	**43.82**	**<0.01**
	**Heatwave or not ×Time**	**0.94**	**0.31**	**3**	**36.52**	**5.48**	**<0.01**
	Log(Total weight)	0.20	0.20	1	12.24	3.54	0.08
Fathead minnow with walleye predator	**Heatwave or not**	**0.37**	**0.37**	**1**	**13.00**	**5.34**	**0.04**
	**Time**	**4.89**	**1.63**	**3**	**42.00**	**23.44**	**<0.01**
	**Heatwave or not ×Time**	**1.87**	**0.62**	**3**	**42.00**	**8.98**	**<0.01**
	**Log(Total weight)**	**0.33**	**0.33**	**1**	**13.00**	**4.69**	**0.05**
Largemouth bass with fathead minnow prey	**Heatwave or not**	**0.65**	**0.65**	**1**	**13.00**	**33.90**	**<0.01**
	**Time**	**4.12**	**1.37**	**3**	**42.00**	**71.16**	**<0.01**
	**Heatwave or not ×Time**	**0.32**	**0.11**	**3**	**42.00**	**5.60**	**<0.01**
	**Log(Total weight)**	**0.49**	**0.49**	**1**	**13.00**	**25.34**	**<0.01**
Walleye with fathead minnow prey	**Heatwave or not**	**0.17**	**0.17**	**1**	**35.00**	**6.18**	**0.02**
	**Time**	**1.14**	**0.38**	**3**	**35.00**	**14.24**	**<0.01**
	**Heatwave or not ×Time**	**0.82**	**0.27**	**3**	**35.00**	**10.23**	**<0.01**
	**Log(Total weight)**	**2.08**	**2.08**	**1**	**35.00**	**77.55**	**<0.01**

The warmwater predator species, largemouth bass, also showed a rapid increase in $\dot{M}O_{2}$ during the 1 h heatwave, but an even higher $\dot{M}O_{2}$ upon encountering fathead minnow, particularly if they had previously experienced a heatwave (Table 2 and Figs 4 and 5). During the heatwave, largemouth bass increased $\dot{M}O_{2}$ by 50% relative to 25°C. This $\dot{M}O_{2}$ during the heatwave was higher than overnight RMR by 60%. The $\dot{M}O_{2}$ (28.71 ± 3.94 mg O_2_ h^-1^) during the heatwave was only 47% of MMR (42.14 ± 7.31 mg O_2_ h^-1^). During the post-heatwave period, the $\dot{M}O_{2}$ of largemouth bass returned to that of RMR. Once fathead minnow were introduced into the holding tank, largemouth bass again showed a significant $\dot{M}O_{2}$ increase, with a 60% increase in $\dot{M}O_{2}$ compared to the “post-heatwave” period for fish not experiencing heatwave before, or double the metabolic rate of fish experiencing the heatwave before. Thus, largemouth bass that experienced the heatwave had higher $\dot{M}O_{2}$ by 40% compared to fish that did not experience the heatwave. Such an increase in $\dot{M}O_{2}$ (20.80 ± 7.37 mg O_2_ h^-1^) took up around 80% of the entire AS (26.23 ± 6.09 mg O_2_ h^-1^).

The coolwater predator species, walleye, also showed a rapid increase in $\dot{M}O_{2}$ during the 1 h heatwave, but did not respond metabolically to prey exposure (Table 2 and Figs 4 and 5). During the heatwave, walleye increased $\dot{M}O_{2}$ by 70% compared to 25°C. This $\dot{M}O_{2}$ during the heatwave was higher than overnight RMR by 70%. The $\dot{M}O_{2}$ (26.60 ± 6.94 mg O_2_ h^-1^) during the heatwave was almost 100% of MMR (24.87 ± 4.27 mg O_2_ h^-1^). During the post-heatwave period, the $\dot{M}O_{2}$ of walleye returned to RMR level. Once fathead minnow were introduced into the holding tank, walleye did not show significant response in terms of $\dot{M}O_{2}$ values relative to post-heatwave. The 48-h mortality rate of walleye used in the analysis was zero.

#### Swimming performance (U_crit_) with heatwaves

**Fig 6:**
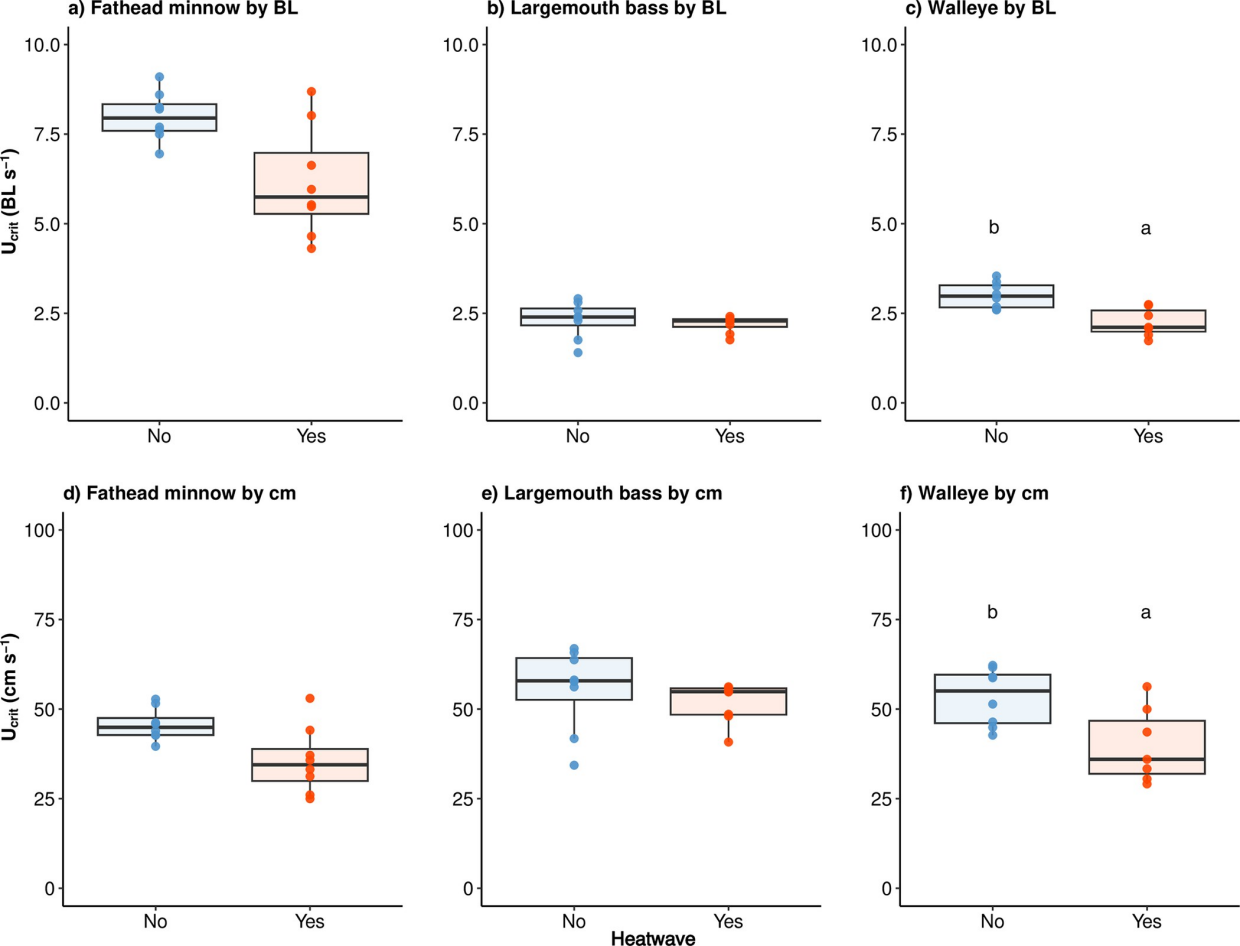
Critical swimming speed (U_crit_, ± se) by body length (BL) or cm of fathead minnow (*Pimephales promelas*) (a, b), largemouth bass (*Micropterus salmoides*) (c, d), and walleye (*Sander vitreus*) (e, f). Measurements occurred after a heatwave or not at 25°C (n = 8 per group). Horizontal line = median; box = first quartile to third quartile; vertical line = 1.5 interquartile range. Different letters represent statistical differences (*p* < 0.05) between heatwave group and control.

Exposure to a short duration heatwave resulted in a difference in swimming performance for warmwater and coolwater fishes examined. More specifically, after experiencing a 1 h 30°C heatwave, fathead minnow did not show a statistically significant change in swimming performance (U_crit_ in BL s^-1^: F_1, 6.9_ = 5.09, *p* = 0.06; U_crit_ in cm s^-1^: F_1, 7.2_ = 4.26, *p* = 0.08) (Table 3 and Fig 6A and 6B). Largemouth bass also showed no change in swimming performance after experiencing the heatwave (U_crit_ in BL s^-1^: F_1, 13.0_ = 0.45, *p* = 0.51; U_crit_ in cm s^-1^: F_1, 13.0_ = 0.74, *p* = 0.41) (Table 3 and Fig 6C and 6D). However, with the heatwave exposures, walleye, the only coolwater species in the study, showed 25% lower U_crit_ (BL s^-1^) (F_1, 11.3_ = 13.40, *p* < 0.01) and approximately 25% lower U_crit_ (cm s^-1^) (F_1, 11.3_ = 9.17, *p* = 0.01) (Table 3 and Fig 6E and 6F).

**Table 3:** Mean (± sd) critical swimming speed (U_crit_) by body length (BL) and cm and body size for fathead minnow (*Pimephales promelas*), largemouth bass (*Micropterus salmoides*), and walleye (*Sander vitreus*).

Species	Treatment	n	Temperature (°C)	Total length (mm)	Total mass (g)	Ucrit (BL s-1)	Ucrit (cm s-1)
Fathead minnow	30°C heatwave	8	25	58 ± 2	1.74 ± 0.24	6.16 ± 1.54	35.68 ± 9.31
	Control	8	25	57 ± 1	1.73 ± 0.24	7.99 ± 0.68	45.68 ± 4.53
Largemouth bass	30°C heatwave	8	25	237 ± 10	201.38 ± 32.20	1.98 ± 0.21	46.77 ± 4.76
	Control	8	25	240 ± 10	222.88 ± 35.11	2.08 ± 0.47	49.75 ± 10.43
Walleye	30°C heatwave	7	25	176 ± 21	47.14 ± 19.40	2.25 ± 0.40	39.83 ± 10.38
	Control	8	25	179 ± 24	55.63 ± 20.32	3.00 ± 0.36	53.37 ± 7.97

## Discussion

Exposure to a short duration heatwave resulted in different physiological responses across the coolwater and warmwater fishes examined. During laboratory simulations of short-duration heatwaves observed in forested streams, the coolwater predator walleye experienced high metabolic cost, low metabolic activity when encountering prey, and reduced swimming performance. All of these are evidence of a thermal disadvantage for walleye relative to sympatric warmwater species that share a similar trophic position in local fish communities. The warmwater predator largemouth bass had relatively high aerobic potential, evidenced by low metabolic cost during the heatwave compared to MMR, increased $\dot{M}O_{2}$ when encountering prey, and consistent swimming performance, even after a 1 h 30°C heatwave. The warmwater prey fathead minnow also showed reduced thermal sensitivity, evidenced by a quick $\dot{M}O_{2}$ response to the heatwave and predator presence. The swimming performance of fathead minnow was slightly affected by the heatwave. Fathead minnow, as a warmwater prey species, overall was less thermally sensitive compared to largemouth bass, but more thermally sensitive compared to walleye.

### SMR, MMR, and AS without heatwaves

Walleye at 25°C showed lower aerobic potential compared to largemouth bass and fathead minnow, implying a competitive disadvantage relative to warmwater fishes in summer. To our knowledge, this is the first study on MMR, SMR, and AS of walleye, using standardized intermittent-flow respirometry. Galarowicz and Wahl [47] previously used closed-vessel respirometry to measure the SMR of walleye, and results were 210–377 mg O_2_ kg^-1^ h^-1^ when acclimated at 25°C. The lower end of their results is close to the SMR of juvenile walleye in this study (Table 1). This is reasonable as the method of closed-vessel respirometry could overestimate SMR because measurements were made right after fish were introduced into the chamber [47], rather than overnight resting values generated in the current study. The mass-specific SMR of the warmwater predator, largemouth bass, was only 37% of walleye. To define whether such differences were due to differences in body size, we referred to [48] and found smaller largemouth bass (79–95 g) still showed lower SMR (i.e., 104–115 O_2_ kg^-1^ h^-1^) at similar temperatures, highlighting the impacts of temperature on $\dot{M}O_{2}$. Besides predators, the prey fathead minnow, for which body weight was < 4% of walleye, showed SMR similar to walleye. This is additional evidence that the coolwater walleye require more energy for homeostasis at the temperatures used in this study, highlighting the thermal sensitivity of walleye under warm summer conditions [49, 50].

The FAS (i.e., 2.1) of walleye was narrower than largemouth bass (i.e., 2.6) and fathead minnow (i.e., 2.9), and all three species had FAS below three. A FAS closer to two has been considered as minimum for digestion due to limited room for specific dynamic action [19, 51]. But FAS is not the only metric to evaluate the aerobic performance of fishes, as oftentimes absolute aerobic scope (MMR–SMR) could be more informative [19]. With similar FAS, walleye clearly showed limited absolute aerobic scope when experiencing heatwaves compared to largemouth bass. In addition, walleye would also have a limited ability to physiologically “multitask” relative to sympatric warmwater predator species, with an impaired ability to concurrently invest energy in growth, reproduction foraging, and escaping predators while at warm water temperatures [21, 52, 53]. With current summer temperatures in the Kaskaskia River Watershed, the competitive disadvantages of the coolwater walleye compared to warmwater largemouth bass is evident physiologically.

### Oxygen consumption rates with the heatwave and predator-prey interactions

Patterns of oxygen consumption following a short duration heatwave differed across the warmwater and coolwater fishes examined in the current study. More specifically during the 1 h heatwave at 30°C, walleye had close to 100% $\dot{M}O_{2}$ of MMR at 25°C, while the mean $\dot{M}O_{2}$ of largemouth bass was only 47% of MMR. This indicates that a 1 h 30°C heatwave, which is already occurring in the Kaskaskia River Watershed [4], could push walleye to their upper aerobic limits, leaving walleye little aerobic potential for activities such as predation and digestion. In addition, the distribution of walleye could be confined to deeper (cooler) areas of larger water bodies, and thermal stressors could also cause elevated oxidative damage, and increased bacterial and parasitic infections, that could reduce fitness [54]. If persisting for extended hours, such heatwaves may eventually cause equilibrium loss or delayed mortality of walleye locally [50], or possible shifts in spatial distributions or altered reproductive events due to warming waters [10].

The same intensity and duration of heatwaves seemed to put little stress on largemouth bass. With the frequency and severity of heatwaves predicted to increase in the near future [54], there could still be thermal safety margins [55] for largemouth bass, but not for coolwater walleye in the Kaskaskia River watershed. This difference in physiological performance could be larger if the heatwaves happen in later spring or early autumn, when the “acclimation” effect would be weaker [56]. In addition to observing metabolic differences across the two predator fish examined, fathead minnow (a small prey fish) had of $\dot{M}O_{2}$ that was 93% of MMR at 25°C, compared to the predator largemouth bass. The difference in the response from different sized warmwater species could represent varying energetic costs during heatwaves, favoring predator species. All of these findings, including the different performance of cool- and warmwater predators, and predators vs. prey species, could influence local community structure in the long term [57] by favoring the performance of warmwater predators that outcompete coolwater predators and influence the abundance of certain prey species.

Beyond quantifying the effects of short-duration heatwaves on the metabolism of individuals, we also incorporated predator-prey interactions using visual and chemosensory stimuli. Hall and Clark [58] witnessed an elevated metabolic cost for prey when noticing the presence of predators, especially when being “attacked” (visual cue) outside of respirometry chambers. Both warmwater species in this study, fathead minnow and largemouth bass, showed a significant metabolic reaction when exposed to their prey or predators. Surprisingly, largemouth bass showed an even higher $\dot{M}O_{2}$ during the prey exposure period compared to when experiencing a 1 h 30°C heatwave. Thus, such heatwaves show limited impacts on their predation capacity. During the testing period, despite not quantifying behavioral responses, anecdotally, fathead minnow showed burst swimming activities within their chambers when exposed to largemouth bass but not when exposed to walleye. Also, when largemouth bass were exposed to their prey, we witnessed a number of predation attempts. In contrast, despite active daily consumption of live fathead minnow during the two-week acclimation period, walleye did not respond metabolically to the introduction of fathead minnow regardless of whether or not they were subjected to the heatwave treatment. Also, when being introduced as predators, fathead minnow did not respond actively to the presence of walleye. These results imply that the reduction in appetite and activity of walleye at or near their upper thermal limit, supporting the “aerobic scope protection” hypothesis [50, 59]. Thus, the limited aerobic potential of walleye following exposure to summer extreme temperatures could inhibit their ability to perform burst swimming or other predation-related activities [21], despite caution should be made here that we did not test another lower acclimation temperature on walleye, to evaluate whether the confinement in respirometry chambers could affect the “normal” appetite. By quantifying the metabolism changes of predators and prey during heatwaves and later interactions, we identified that coolwater walleye were potentially unable to have enough aerobic potential to safely overcome heatwaves and generate effective predation responses.

### Swimming performance under summer conditions following heatwaves

Largemouth bass showed no reduction in U_crit_ after the 1 h 30°C heatwave, remaining around 2 BL s^-1^ at 25°C. This is similar to previous tests of 2.17 ± 0.12 BL s^-1^ at 25°C [38]. Fathead minnow showed a noticeable, although not significant, reduction in swimming performance after the heatwave, suggesting a reduced predator-avoidance capacity after heatwaves, especially when considered in comparison to thermally-tolerant predators like largemouth bass. The U_crit_ of fathead minnow at 25°C is close to previous reports (i.e., 45.68 ± 4.53 vs. ~ 38 cm s^-1^) [60]. Unlike warmwater predator, the swimming performance of coolwater walleye was significantly impaired following a 1 h 30°C heatwave, with a ≥ 25% decrease in U_crit_ (both in BL and cm). Such an impairment in swimming further confirms the risk of narrow AS, as swimming is largely fueled by aerobic metabolism [61]. This study is the first to quantify the swimming performance of walleye at 25°C, close to its chronic upper thermal tolerance [36]. It is reported that under lower temperature, 20.5°C, the U_crit_ of walleye (fork length = 180 mm) was projected to be 0.35 and 0.64 cm s^-1^ with water velocity increasing 10 cm s^-1^ every 60-min and 10-min period, respectively [62]. With similar total length to largemouth bass, walleye would be expected to have larger U_crit_ due to its higher swimming potential in streams and rivers [62]. However, the advantage of swimming performance of walleye could largely vanish under heatwaves and elevated summer temperatures, leaving largemouth bass, or other warmwater predators, to be a better competitor in riverine environments [63], where swimming is important for maintaining position, movement, attacking, and escape [38, 61].

Our results offer higher-resolution guidance for future stream flow management for walleye after considering not only static but also fluctuating summer temperatures. Researchers and practitioners have long known that management activities, such as protection of riparian areas and preservation of thermal refugia, can mitigate the impacts of elevated temperatures fishes [64]. However, results from the current series of experiments not only underscore the importance of considering heatwaves in management activities, but also highlight that the impacts of heatwaves will vary across thermal guilds of fishes and can contribute to community or populations-levels shifts. For example, the current data demonstrate that even short-duration increases in temperature can result in differences in metabolic costs and predator-prey interactions for Midwestern, riverine fishes, providing additional justification for protecting riparian areas, enhancing thermal refuges or stocking of thermally tolerant prey in an effort to preserve communities and fish assemblages [64, 65]. Also, for other coolwater or coldwater species, swimming performance can be impaired not only at constant elevated temperatures, but also from short duration heatwaves, and should be considered when designing migration paths or corridors. We encourage others to continue studies of how heat waves can impact individual fish, as well as fish communities, by conducting subsequent studies that include actual predator/prey (feeding) studies, work with other species of fish, along with studies that have stronger links to fitness, including responses such as reproductive output and offspring survival in an effort to better quantify the potential impacts of these events. Individuals used in this study were also juveniles (sub-adults), and similar work in the future should be performed on larger (adult) fish that have spent more time in the wild and may have a different aerobic thermal window than the juveniles used in this study [66].

## Conclusions

Recent modeling work using recruitment and abundance data, coupled with abiotic projections under climate change, has projected shifts from coolwater walleye to warmwater largemouth bass in Midwestern lakes [12]. We sought to use ecophysiology to define possible mechanisms related to these community shifts, focusing on metabolic and swimming parameters. Our results show that walleye, and possibly other coolwater species, will experience a competitive disadvantage relative to warmwater species like largemouth bass following short-duration heatwaves, which could be contribute to a loss of native coolwater fishes at the southern edge of their range [12, 67]. What is more, other stresses, such as low dissolved oxygen and high ammonia, often coexist with heatwaves and could further impact the more sensitive coolwater species [68]. By incorporating such physiological understanding into modeling of biodiversity [69, 70], we could more efficiently predict the impacts of climate change, then offer conservation guidance for coolwater species (e.g., walleye and Salmonid species) that are economically and spiritually important [71, 72].
